# Supplementary material for: A leaf reflectance-based crop yield modeling in Northwest Ethiopia
Source: PLoS One. 2022 Jun 16;17(6):e0269791. doi: 10.1371/journal.pone.0269791 (PMC9202864; doi:10.1371/journal.pone.0269791)
Supplement: Attachment — Submitted filename: Reviewers comments.docx [file pone.0269791.s005.docx]

I found some deficiencies in the way the article is written. The authors did not specify the experimental design used. Data collection protocols are not described in sufficient details. For example, Fig. 4 shows the spectral data being taken on leaves laid out on a ‘bench/table’ but in the text there is no mention of destructive (Fig 4b and c) sampling. Why the authors chose destructive sampling is not justified in the article. I would expect the researchers to collect spectral data *in-situ* in the field. However, whatever the method used should have been spelt out clearly and provide justification. How did they control the background noise? I do not see any value whatsoever in showing readers a heap of harvested maize stover (Fig. 4d) and ear/cob images (Fig.4e, unless there was cob/ear imaging as part of data collection, which I did not see in the article). Averaging spectral signatures of different treatments is not advisable (Fig. 5) unless the authors found no significant differences across treatments (which should be stated). Otherwise, I see no value in Fig 5. The authors included supplementary data (S1 & S2) but this is not mentioned anyway in the article except Table notations (L403-405). I recommend major revision.

Some specific comments:

Abstract

- Revise L20 and remove the word ‘was’
- Insert the letter ‘L.’ immediately after the word ‘*mays’* L21
- Abstract is a stand alone so must be concisely written with methodologies used spelt out clearly precisely. This is lacking in this manuscript.

Introduction

- Revise L41
- The sentence (L52-53) ‘…examined …’ need to be revised.

Materials and Methods

The methodology is not described in sufficient detail.

- L62 insert the word ‘from’ before 1994-2021.
- L79 … ‘geographically identified’… What does this mean?
- L81 The authors need to specify the number of rows per each 12x12 plot, the maize variety grown,
- L82 …. What are traditional methods?
- L3 … When was the fertilizer applied?
- L86 – delete the word ‘was’
- L88 …. ‘on days’ – specify the days or delete these two words.
- L89 …what is outdoor solar?
- L90 … the statement is hanging.
- L94-95. I do not see the value of Figure 4d ‘maize heap’ in this article
- L98 .. the authors mentioned ‘novel’ SVIs but the Table 1 show the traditional SVI!
- L103 Five plants were harvested. How? From which row? When (days after planting/phenological stage)?
- L106 The authors should use the standard terms used in scientific writing. For example, instead of ‘grain water’ use ‘grain moisture’.
- L110 replace the word ‘under’ with ‘in’ or ‘using’
- L120-124 may be misplaced.

Results and discussion

- Figure 5 shows the spectral signature of maize. At what phenological stage of the maze was this measured. From M&M, 24 plots were planted with or without soil bund and at 3 different slopes. Does Fig. 5 represent the average of all the different treatments? What value does Fig. 5 add to the manuscript if it is an average across treatments? I would expect the authors to state concisely what was measured, when and a comparison of the different treatment used in the experiment.
- L147. What are ‘hybrids of bands’ (needs to be explained explicitly for readers to understand what the authors are trying to convey)? Does this mean a combination of bands?
- Results displayed in Figs 6-7 need to be well explained and discussed. This is lacking in L138-151

Overall, the article requires major revision to be accepted for publication.
