# Supplementary material for: A leaf reflectance-based crop yield modeling in Northwest Ethiopia
Source: PLoS One. 2022 Jun 16;17(6):e0269791. doi: 10.1371/journal.pone.0269791 (PMC9202864; doi:10.1371/journal.pone.0269791)
Supplement: Attachment — Submitted filename: Response to Reviewers.docx [file pone.0269791.s006.docx]

Date: June 01, 2022

**Rebuttal letter**

PONE-D-22-01589R1
We are happy about the academic editor and the reviewers’ comments, which strengthen the current version of the manuscript **“A leaf reflectance-based crop yield modeling in Northwest Ethiopia”.** In addition, our supreme sincere gratitude goes to you and the reviewers who devote their valuable time to bring our manuscript to a competent paper.

We have provided a one by one reply to all concerns and comments given below. We thank you for your consideration of this resubmission and look forward to your response.

Best regards,

Gizachew Ayalew Tiruneh (on behalf of all co-authors)

Lecturer in Debre Tabor University

Ph.D. Fellow in soil science, Bahir Dar University

Email: tiruneh1972@gmail.com

Dear editor and reviewers, thank you so much for taking your valuable time to elevate the quality of our manuscript. We do hope that the editor’s and Reviewer’s concerns will be addressed.

**Editor comments:**

**Comment 1:** A rebuttal letter that responds to each point raised by the academic editor and reviewer(s). You should upload this letter as a separate file labeled 'Response to Reviewers'.

Response: We addressed the concerns provided by the editor and reviewers and uploaded a file labeled “Response to Reviewers”.

**Comment 2:** A marked-up copy of your manuscript that highlights changes made to the original version. You should upload this as a separate file labeled 'Revised Manuscript with Track Changes'.

Response: We tried to highlight our revised paper with tracked changes. We uploaded this as a separate file labeled 'Tracked changes'.

**Comment 3:** An unmarked version of your revised paper without tracked changes. You should upload this as a separate file labeled 'Manuscript'.

Response: We revised our manuscript without tracked changes. We uploaded this as a separate file labeled 'Manuscript'.

**Comments 4**: If you would like to make changes to your financial disclosure, please include your updated statement in your cover letter.

Response: We have not made any changes to financial disclosure.

**Comments 5:** If applicable, we recommend that you deposit your laboratory protocols in [protocols.io](http://www.google.com/url?q=http%3A%2F%2Fprotocols.io&sa=D&sntz=1&usg=AOvVaw1BDT1QMuwApptyZbx7v6P7) to enhance the reproducibility of your results. Protocols.io assigns your protocol its own identifier (DOI) so that it can be cited independently in the future. For instructions see: [https://journals.plos.org/plosone/s/submission-guidelines#loc-laboratory-protocols](https://www.google.com/url?q=https%3A%2F%2Fjournals.plos.org%2Fplosone%2Fs%2Fsubmission-guidelines%23loc-laboratory-protocols&sa=D&sntz=1&usg=AOvVaw3u6_rcoIhAhM0XlajZ1VSM). Additionally, PLOS ONE offers an option for publishing peer-reviewed Lab Protocol articles, which describe protocols hosted on [protocols.io](http://www.google.com/url?q=http%3A%2F%2Fprotocols.io&sa=D&sntz=1&usg=AOvVaw1BDT1QMuwApptyZbx7v6P7). Read more information on sharing protocols at [https://plos.org/protocols?utm_medium=editorial-email&utm_source=authorletters&utm_campaign=protocols](https://www.google.com/url?q=https%3A%2F%2Fplos.org%2Fprotocols%3Futm_medium%3Deditorial-email%26utm_source%3Dauthorletters%26utm_campaign%3Dprotocols&sa=D&sntz=1&usg=AOvVaw03onmnAbA9-D2bKW5pbLYQ)

Response: Not applicable.

**Comments 6:** Please review your reference list to ensure that it is complete and correct. If you have cited papers that have been retracted, please include the rationale for doing so in the manuscript text, or remove these references and replace them with relevant current references. Any changes to the reference list should be mentioned in the rebuttal letter that accompanies your revised manuscript. If you need to cite a retracted article, indicate the article’s retracted status in the References list and also include a citation and full reference for the retraction notice.

[Note: HTML markup is below. Please do not edit.]

Response: Thank you for your advice. We have checked that all references in the text are also in the reference and vice versa and all are complete and correct. We do not have retracted papers.

Reviewers' comments to Questions

**Comments to the Author**
**Comment 1.** If the authors have adequately addressed your comments raised in a previous round of review and you feel that this manuscript is now acceptable for publication, you may indicate that here to bypass the “Comments to the Author” section, enter your conflict of interest statement in the “Confidential to Editor” section, and submit your "Accept" recommendation.

Reviewer #1: All comments have been addressed

Reviewer #2: (No Response)

**Response:** Thank you the reviewer for your feedback.

**Comment 2.** Is the manuscript technically sound, and do the data support the conclusions?

The manuscript must describe a technically sound piece of scientific research with data that supports the conclusions. Experiments must have been conducted rigorously, with appropriate controls, replication, and sample sizes. The conclusions must be drawn appropriately based on the data presented.

Reviewer #1: Yes

Reviewer #2: Yes

**Response:** Thank you.

**Comment 3:** Has the statistical analysis been performed appropriately and rigorously?

Reviewer #1: Yes

Reviewer #2: No

Thank you. We have gone thoroughly the revised manuscript, and hopefully that the reviewers will be satisfied.

Comment 4. Have the authors made all data underlying the findings in their manuscript fully available?

The [PLOS Data policy](http://www.google.com/url?q=http%3A%2F%2Fwww.plosone.org%2Fstatic%2Fpolicies.action%23sharing&sa=D&sntz=1&usg=AOvVaw0txrLazDj_2hFfsX8TT2cP) requires authors to make all data underlying the findings described in their manuscript fully available without restriction, with rare exception (please refer to the Data Availability Statement in the manuscript PDF file). The data should be provided as part of the manuscript or its supporting information, or deposited to a public repository. For example, in addition to summary statistics, the data points behind means, medians and variance measures should be available. If there are restrictions on publicly sharing data—e.g. participant privacy or use of data from a third party—those must be specified.

Reviewer #1: Yes

Reviewer #2: Yes

**Response:** Thank you.

**Comment 5.** Is the manuscript presented in an intelligible fashion and written in standard English?

PLOS ONE does not copyedit accepted manuscripts, so the language in submitted articles must be clear, correct, and unambiguous. Any typographical or grammatical errors should be corrected at revision, so please note any specific errors here.

Reviewer #1: Yes

Reviewer #2: No

Response: Thank you for your advice. We have thoroughly revised our manuscript with the help of Grammarly (premium) and licensed iThenticate software (as attached document), and we do hope that the reviewers concerns will be addressed.

Review Comments to the Author

Please use the space provided to explain your answers to the questions above. You may also include additional comments for the author, including concerns about dual publication, research ethics, or publication ethics. (Please upload your review as an attachment if it exceeds 20,000 characters)

**Comments of Reviewer #1:** Introduction
Page 3 line 45: Replace 'time taking' with 'time consuming'
Page 3 line 48 and 49: The two sentences on these lines begin with 'As a result...' consider revising one of the sentences.

Generally, the motivation in the introduction is still weak. The motivation should be strengthened.

**Response:** Thank you. We appreciate your valuable comments. We tried to address the comments and incorporated them in the revised manuscript in the introduction section. We hope that this revised version will be satisfying.

**Reviewer #2: Comments**
Comment 1: Summary
The authors made partial corrections suggested in the first round of review. The manuscript still lacks details, mainly in the following areas: motivation/rationale in the introduction; English editing; clarity in materials and methods; further analysis using a combination of VIs in the model; referencing latest publications on yield modeling using remote sensing; and detailed discussion of results. Specific comments are given below. I recommend further revision.

Abstract
The last concluding statement (L35-37) talks about policy makers effectively managing resources! How?
Key words:
Why is “Arid” included as a key word when it is not mentioned anyway in the manuscript body except authors’ address and references? The study area receives rainfall ranging from 1,076 to 1,953 mm and cannot be classified as arid.

**Response:** Thank you for your valuable comments. We tried to address the comments and incorporated them in the revised manuscript in the abstract, introduction, materials and methods, combining of VIs in the model; referencing latest publications on yield modeling using remote sensing; and detailed discussion of results. Besides, we have thoroughly revised our manuscript with the help of Grammarly (premium) and licensed iThenticate software (as attached document), and we do hope that the reviewers concerns will be addressed.

Comment 2: Introduction
The introduction needs to be revised. The motivation/rationale is still weak or not convincing.
L49-51 should be taken to the second last paragraph of introduction and remove “As a result” from the statement.
L61-62 need rephrasing and qualifying.
L64 remove “as essential devices”
L75-76 remove “examined to”
L77, authors need to define the term “bund” for readers who are not familiar with the term/system

**Response:** Thank you for your comments. We tried to address the above comments and incorporated them in the revised manuscript including in the introduction section, and we do hope that the reviewers concerns will be addressed.

**Comment 3.** Material and Methods
The authors mentioned “midland”. This is not the right way to classify based on altitude. Better to say mid-altitude. “Midland” may mean a different thing altogether.
L86, the word “Teff” should not be in italics
The slope category is overlapping (L92-93): gentle (2–5 %), sloping (5–10 %), and strongly sloping (10–15 %). Please correct. I also suggest the mid slope (5-10%) category to be referred to as “medium” sloping rather than just “sloping” with corrections on overlapping categories.
Paragraph L97-102 lacks clarity on what was done exactly. The authors mentioned crop sampling points and planting. Please give precise details of what exactly was done in a precise and consistent manner. In other words, this section should provide readers with enough detail to replicate the study.
The hybrid name should be fully specified (L99). I suspect the full name is “BH540”!
L100 talks about traditional methods used for pest control. These need to be specified since they are not standard throughout the world.
Figures 2a and 2d are not referred to in the text.
L114 – statement which starts “Sun radiation…” needs to be rephrased.
L121, remove “can” and add “s” to the word “show”
L124, Table 1 remove “Explanation of the”
L128 replace “balancing” with “weighing”. Put unit of measurement after the word “plant” before converting to t/ha. Remove “gauging it with a balance”.
L129 Replace “conveying” with converting.
L131 Remove or rephrase “was articulated as a ton/ha”
L136 remove “values of”

**Response:** Thank you for your suggestion. We tried to address the above comments and incorporated them in the revised manuscript including in the Material and Methods section, and we do hope that the reviewers concerns will be addressed.

**Comment 4.** Results and discussion
Most of the results were not discussed. Better to separate “Results” and “Discussions”. It would have helped and strengthen the manuscript if the authors do a combination of spectral bands on their own, VIs own their own and bands and VIs in prediction. See comment below (L218). For recent work on combining bands and/or VIs see: [https://www.sciencedirect.com/science/article/pii/S0168169921001460](https://www.google.com/url?q=https%3A%2F%2Fwww.sciencedirect.com%2Fscience%2Farticle%2Fpii%2FS0168169921001460&sa=D&sntz=1&usg=AOvVaw06uJiwNVwzGwFzY2Owdp4Y)
L148-150 which starts with “All soil …”. Where is this statement coming from and it is referenced?
L150-152 – Why quoting references when the authors are reporting their results? This can only be done when discussing! This also applies L175-177.
L156 Put an "=" sign between Min and minimum and the same applies to max and maximum (table legend)
L179-180 needs to be rephrased.
L194-195 remove “in the planting period”
L209 Start this statement as "The different relationships among SVIs and maize GY recorded (Fig 5 -6) could...."
L215 replace “bases” with “based”
L216-217 rephrase
L218 - Why did the authors not combine spectral bands and VIs and assess their predictive power rather than referring to literature? In their manuscripts, the authors used VIs as individual input variables to the model yet recent studies suggest that predictions can be improved when using a combination of bands or VIs in the model (see recent publication in Computers and Electronics in Agriculture journal): [https://www.sciencedirect.com/science/article/pii/S0168169921001460](https://www.google.com/url?q=https%3A%2F%2Fwww.sciencedirect.com%2Fscience%2Farticle%2Fpii%2FS0168169921001460&sa=D&sntz=1&usg=AOvVaw06uJiwNVwzGwFzY2Owdp4Y)
L218 – Please quote recent work – e.g.: [https://www.sciencedirect.com/science/article/pii/S0168169921001460](https://www.google.com/url?q=https%3A%2F%2Fwww.sciencedirect.com%2Fscience%2Farticle%2Fpii%2FS0168169921001460&sa=D&sntz=1&usg=AOvVaw06uJiwNVwzGwFzY2Owdp4Y)
L226 rephrase statement which begins “NDVI…”
L231 – give examples of “various factors”
L273 - The "red lines" inserted in the variable importance is not explained what it represents or serves. Otherwise, the two figures (r and s) are informative enough without the lines unless the authors want to show extra information depicted by the lines, which needs to be explained in the text or figure caption.
L289-290 Rephrase
L290-293 - This statement is not clear. How does predicting yield (a quantitative measure) has to do with time of harvesting? How do you locate any biotic and abiotic stresses through yield prediction? Rather you can be able to explain yield variation if you know the prevailing stresses and soil type.
L294 - Table 5 is not mentioned in the text. Also put a demarcating line before Mean.
For Use “LSD” instead of “MSD” and indicate at what p-value.
L299 replace “various” with “different”
L300 Add p-value for significant and not significantly immediately after the words “markedly” and “substantially”, respectively
L300 Put appropriate p-value. As it is, it means it was highly significant. It should be p > xx for example.
L306 what is the purpose of term “Tukey, p < 0.0001” here?

**Response:** Thank you for your suggested reference. We tried to address the above comments and incorporated them in the revised manuscript including in the Results and discussion section, and we do hope that the reviewers concerns will be addressed.

**Comment 5.** Conclusion
L314 replace “over with “in”
L315 Remove “novel” - these are not new VIs
L315 – statement beginning “However, ….” needs to be qualified by stating".... using our data" at the end of the statement
L318 The statement which begins “We obtained ..” is hanging.
L324-326 How? Not clear. Please explain clearly.
L326-328 Rephrase this recommendation. Studies on yield prediction have already been done at different phenological stages for certain stresses, e.g.: [https://www.sciencedirect.com/science/article/pii/S0168169921001460](https://www.google.com/url?q=https%3A%2F%2Fwww.sciencedirect.com%2Fscience%2Farticle%2Fpii%2FS0168169921001460&sa=D&sntz=1&usg=AOvVaw06uJiwNVwzGwFzY2Owdp4Y)

**Response:** Thank you for your comments and suggested reference. We tried to address the above comments and incorporated them in the revised manuscript including in the Conclusion section, and we do hope that the reviewers concerns will be addressed.

**Comment** 6. PLOS authors have the option to publish the peer review history of their article ([what does this mean?](https://www.google.com/url?q=https%3A%2F%2Fjournals.plos.org%2Fplosone%2Fs%2Feditorial-and-peer-review-process%23loc-peer-review-history&sa=D&sntz=1&usg=AOvVaw2K3Sj0-NOk-Ks9rGu0Ac8e)). If published, this will include your full peer review and any attached files.


If you choose “no”, your identity will remain anonymous but your review may still be made public.


**Do you want your identity to be public for this peer review?** For information about this choice, including consent withdrawal, please see our [Privacy Policy](https://www.google.com/url?q=https%3A%2F%2Fwww.plos.org%2Fprivacy-policy&sa=D&sntz=1&usg=AOvVaw06zP6_FDUktleQDUz7rvy1).

Reviewer #1: No

Reviewer #2: No

[NOTE: If reviewer comments were submitted as an attachment file, they will be attached to this email and accessible via the submission site. Please log into your account, locate the manuscript record, and check for the action link "View Attachments". If this link does not appear, there are no attachment files.]

While revising your submission, please upload your figure files to the Preflight Analysis and Conversion Engine (PACE) digital diagnostic tool, [https://pacev2.apexcovantage.com/](https://www.google.com/url?q=https%3A%2F%2Fpacev2.apexcovantage.com%2F&sa=D&sntz=1&usg=AOvVaw26riLzallEMy_QMYCA6Z8Y). PACE helps ensure that figures meet PLOS requirements. To use PACE, you must first register as a user. Registration is free. Then, login and navigate to the UPLOAD tab, where you will find detailed instructions on how to use the tool. If you encounter any issues or have any questions when using PACE, please email PLOS at [figures@plos.org](https://mail.google.com/mail/u/0/h/spjl8r6q9r4q/?&cs=wh&v=b&to=figures@plos.org). Please note that Supporting Information files do not need this step.

Response: Thank you. We have used PACE with this submission, so this should be right.

Please note that once again, thank you very much. Your comments are greatly appreciated.

Best regards,

Gizachew Ayalew Tiruneh (on behalf of all co-authors)

Lecturer in Debre Tabor University

Ph.D. Fellow in soil science, Bahir Dar University

Email: tiruneh1972@gmail.com
