# Supplementary material for: A leaf reflectance-based crop yield modeling in Northwest Ethiopia
Source: PLoS One. 2022 Jun 16;17(6):e0269791. doi: 10.1371/journal.pone.0269791 (PMC9202864; doi:10.1371/journal.pone.0269791)
Supplement: Attachment — Submitted filename: Reviewer Comments.docx [file pone.0269791.s004.docx]

**Reviewer Comments**

**General comments**

1**.** The introduction section should be re-written, it lacks depth. Currently, it sounds like highlights of a paper. The authors should motivate on the relevance of their study in improving food security. The objective (s) of the study are not adequately conceptualized in the introduction. The following should be included in this section

- General overview of the state of agriculture and food security in Ethiopia (not a few random statistics)
- Give a brief explanation of why crop yield estimation is important in agriculture/food security? Why should we do it? If there is no adequate justification on this issue, the study won’t be necessary
- Give an account of the old/traditional methods that were being used to estimate crop yields…what are their shortcomings/weaknesses ---- at this point remote sensing comes in
- Introduce remote sensing, how does it address the weakness presented by traditional methods in crop yield estimation - Mention previous works (hyperspectral remote sensing preferably, since you are using a Spectroradiometer).
- Introduce spectral vegetation indices, what are their significance in building crop yield estimation models?
- Now that we know remote sensing has been previously used in crop yield estimation, please highlight the gap that you would like to address – what new information do you wish to generate?

2. The slope gradients on the experimental design are overlapping. If gently sloping is 2-5% and 5-10% is slopping then how would we classify a slope with 5%, which category does it fit in?

3. Figure 3 is not adding any value to the manuscript. The focus is not on the ASD but crop yield modeling. I suggest you remove figure 3.

4. There is a lot of relevant information missing in the methodology section;

- ‘Five maize plants were harvested at each plot and air-dried for a week’. – How many plots did you have?
- water content of 12 % - how did we get to the 12% water content. Give a scientific explanation/justification, add references as well.
- There are many spectral vegetation indices used in remote sensing studies. However, the current study chose six indices. How did you choose them? What is the justification for using those six indices?

5. Please revise the manuscript, there are a lot of abbreviations that were never written in full, e.g. IBM, SPSS. All abbreviations should be written in full the first time they appear in the manuscript.

6. ‘Fig. 5 shows the spectral response of the maize crop between the visible and SWIR (350- 128 2500 nm) bands over the growing season’ The authors mention that the spectral response curve was over the growing season. However, the explanation in the results section does not specify the phenological stage at which the spectral reflectance was measured. If the spectral measurements were recorded over the growing season as expected, then we expect to see spectral response curves at each growth stage (not sure how many for maize).

**Specific comments**

Page 2 line 20: ‘This study was used leaf reflectance from…’ The statement is grammatically incorrect, please revise.

Page 5 line 88: ‘…(USA) on days between 10:00 and 11:00 a.m.’ which days are you referring to? The statement is not clear.

Page 6, Table 1: When citing references for vegetation indices use the original references (the person who originally developed the index).

Page 10 Line 174: ‘The importance of the predictor variables…’ Please revise this statement
